# Integration of gene expression data with prior knowledge for network analysis and validation

**DOI:** 10.1186/1756-0500-4-520

**Published:** 2011-11-28

**Authors:** Michael Ante, Edgar Wingender, Mathias Fuchs

**Affiliations:** 1 Department of Bioinformatics, Medical School, Georg-August-University Goettingen, Goldschmidtstr. 1, 37077 Goettingen, Germany

## Abstract

**Background:**

Reconstruction of protein-protein interaction or metabolic networks based on expression data often involves in silico predictions, while on the other hand, there are unspecific networks of in vivo interactions derived from knowledge bases.

We analyze networks designed to come as close as possible to data measured in vivo, both with respect to the set of nodes which were taken to be expressed in experiment as well as with respect to the interactions between them which were taken from manually curated databases

**Results:**

A signaling network derived from the TRANSPATH database and a metabolic network derived from KEGG LIGAND are each filtered onto expression data from breast cancer (SAGE) considering different levels of restrictiveness in edge and vertex selection.

We perform several validation steps, in particular we define pathway over-representation tests based on refined null models to recover functional modules. The prominent role of the spindle checkpoint-related pathways in breast cancer is exhibited. High-ranking key nodes cluster in functional groups retrieved from literature. Results are consistent between several functional and topological analyses and between signaling and metabolic aspects.

**Conclusions:**

This construction involved as a crucial step the passage to a mammalian protein identifier format as well as to a reaction-based semantics of metabolism. This yielded good connectivity but also led to the need to perform benchmark tests to exclude loss of essential information. Such validation, albeit tedious due to limitations of existing methods, turned out to be informative, and in particular provided biological insights as well as information on the degrees of coherence of the networks despite fragmentation of experimental data.

Key node analysis exploited the networks for potentially interesting proteins in view of drug target prediction.

## Background

The structure of cell signaling is governed by complex patterns of interaction. In the past decades, great progress toward understanding networks of protein interactions and the metabolic flow of matter has been made. There are many ways of computational network reconstruction, differing fundamentally in the nature of the underlying data's organization and interpretation. Networks derived from single gene expression profiles often take advantage of sophisticated mathematical methods, thus providing a wealth of information on actual biological conditions. However, they necessarily involve prediction of interactions. On the other hand, semantically controlled extraction of database cross-sections leads to networks based on carefully organized knowledge gathered in a great many in vitro and in vivo experiments. Hence, the static nature of databases may be contrasted with the snapshot nature of gene expression data. Reconciling these different approaches is important for obtaining a unified view on gene expression.

There is vast literature on network inference from expression data (see for instance [1-9]).

Since databases contain little tissue- or disease-specific information, they are concerned with a theoretical whole genome-stem cell. (In a few cases, taxa- or species-specific networks have been reconstructed though [10,11].) Furthermore, such theoretically derived networks do not allow for definition of environmental or histological conditions or time-dependent processes such as signal-triggered events. Hence, it is often impossible to determine if a given set of reactions with matching substrates gives a biologically plausible chain. In particular, in database-derived networks molecules can be connected although they are in no tissue simultaneously highly expressed.

Both methods of network inference, the one based on expression time-series and the other on databases carry a high probability of inferring undesirable links. Thus, tissue- or disease-related information is concealed in plenty of unspecific data. Several approaches to link both methods have been proposed. However, this has often been done on an ad hoc-basis, taking knowledge only for selected molecules into account [12,13]. A concept for the extraction of tissue- and cell type-specific transcription factor-gene networks based on EST abundance of transcription factor-encoding genes has been proposed recently [14].

It is the goal of the present paper to set up networks whose nodes are expressed in a single tissue while the interactions are taken from prior knowledge, contained in manually curated databases. We thus aim at coming as close as possible to data measured in vivo by excluding any prediction in network inference. Precisely, we filter the reference networks defined in [15] onto expression data from breast cancer tissue samples and analyze the results. Specifically, we map the detectable expression set's genes onto database-derived networks and prune the latter by retaining only those vertices which are mapped to detectable genes and their edges. A second filtering method includes the shell defined by the latter network's 1-neighborhood in the complete network. These two methods are called strict and 1-extended, respectively.

The reference networks' protein identifier format is abstracted in order to be coarse enough to identify very similar proteins. On the one hand, this leads to good network connectivity properties, on the other hand, this implies the need to perform "benchmark tests" for network validation. Thus, we pay particular attention to assessing the degree to which this coarseness leads to information loss; in fact, we show that tissue-specificity properties are retained and that, similarly, the suspicious pathways are over-represented. This is done by performing statistical hypothesis tests against refined null models; these become necessary because quite often common null models are biologically unfeasible. The next step is to extract new information from the filtered networks. Precisely, we analyze the networks for key nodes and show that this yields information which fits well to the findings of the pathway over-representation analysis in the sense that both analyses often hint independently at the same or closely related underlying molecular mechanisms. In particular, these mechanisms turn out to be those around cell cycle regulation.

The databases we rely upon are the TRANSPATH database on signal transduction [16] and the KEGG biochemical database [10].

## Methods

### Overview

**Figure 1:**
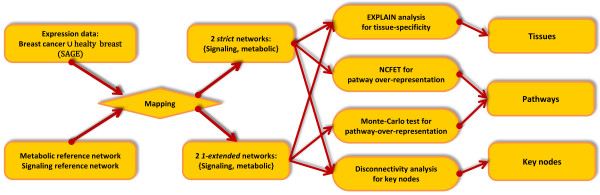
**Workflow scheme for the analysis of the filtered networks**. We obtain publicly available expression sets from breast cancer data (CGAP SAGE libraries); these are mapped to TRANSPATH signaling molecules and KEGG metabolic activities. Strict resp. 1-extended filtering yields 4 networks per disease condition.

The workflow is depicted in Figure 1. We associated to a fixed reference network and to several gene expression sets sub-networks of the reference network by means of mapping the expression data on the reference network's vertices. The procedure's potential to yield tissue-specific information on biological functions was assessed, involving tissue-specificity analysis, pathway analysis, and key node analysis. We propose modified methods for assessment of pathway over-representation whereas the key node algorithms and tissue-specificity checks we employed have been published.

### Data acquisition

#### Network extraction

The reference network used in our study was retrieved from the TRANSPATH database (release 2008.4) by extracting all semantic molecular reactions which take part in signal transduction. The network's vertices are proteins with signaling function and small signaling molecules while edges are created by the semantic syntax as described in [17] and used in [15], yielding a network with 1843 vertices and 3931 edges with a largest strongly connected component of 328 vertices, 454 in the bow-tie-analysis' in-component, and 546 in the out-component [18]. All information available on orthologous (in most cases, mouse, human and rat) proteins and their reactions was merged, giving rise to the so-called ortho-level of gene identifiers. Similarly, information about tissue- or cell type-specificity was ignored. We studied the molecular networks defined by leaving out information on transcription regulation, in contrast to the (usually larger) gene regulatory networks. The reason for focusing on the former is to ensure network consistency, i.e. to stay within the same level of molecular biology and on the narrower time-scale of mere protein-protein interaction; moreover, there is considerable uncertainty about the complete target pool of a single or even the compound of several transcription factors.

The KEGG-LIGAND-based (release 40.0) metabolic reference network was designed in an enzyme-centered way in order to be comparable with the protein-centered view of the TRANSPATH-derived network. Each node is an EC number representing a metabolic enzyme activity with edges standing for a common substrate which is the product of the reaction catalyzed by the enzyme on the tail and the educt of the reaction catalyzed by the enzyme on the head. Substrates such as ATP or water taking part in more than 100 reactions were discarded. This is the gene-family equivalent to the network extraction method presented and used in [19]. The metabolic reference network is included in Additional File 1.

#### Expression data

Breast cancer expression data were downloaded from NCBI CGAP database using the Sage Digital Gene Expression Displayer (DGED) [20]. We considered the union of normal and disease tissue, thus retaining silenced or induced genes for consideration.

#### Mapping

The SAGE data's UNIGENE identifiers were mapped on TRANSPATH, resp. KEGG, gene entries and subsequently to the encoded proteins as well as restricted to those which are contained in the reference network. Due to the fact that this new network's vertex set only consists of those vertices of the reference network that reflect the expression data, the corresponding subnetwork of the reference network was called strictly filtered. Additionally, we considered for each such subset of the reference graph a larger variant, called 1-extended filtering, which includes all nodes adjacent by an edge in either direction to a node in the strict subset. This graph includes more prior knowledge since it is slightly closer to the reference network.

### Global network analysis

#### ExPlain

Functional analysis is done with ExPlain 3.0 (BIOBASE GmbH, Germany) [21] to provide further information on tissue-specificity and disease marker over-representation. In this context, an advantage of the ExPlain tool is that no further information loss by gene format mapping occurs since the signaling reference graph is already given in TRANSPATH identifier format. These identifiers are internally mapped by ExPlain onto species-specific level from human. Nevertheless this analysis is used to tell how much information is already present on the ortho-level. Internal FDR control methods were used throughout (see below for general information on the FDR).

#### Pathway over-representation

In order to exhibit those TRANSPATH resp. KEGG pathways which are differentially expressed with respect to all other tissues than the one of the probe's origin.

In order to integrate quantitative information, we pursued an approach based on the BioGPS expression data U133A/GNF1H [http://biogps.gnf.org/downloads/, download date 05/17/2010, [22]], which provides tissue-specific information in the form of a matrix *A *where *A_ij _*is the GCRMA-normalized intensity of gene spot *i *for tissue *j*. We used the BioGPS matrix as is because none of the 84 tissues is super- or subordinate to lactiferous duct. The overall expression value *E*_i _of spot *i *was then taken to be the sum Σ*_j _A_ij _*over all these 84 in the data set, and the drawing probability for the reference network entry that corresponds to spot *i *was set to be the ratio *E_i _*/Σ*_n _E_n _*of the latter value to the sum over all rows which correspond to such gene identifiers that could be mapped to the reference graph, where "average" corresponds to the pathway resp. non-pathway but mappable group. Thus, the null hypothesis was that the subset of the reference graph's vertices was chosen from a gene pool where the amount of a certain gene corresponds to its expression in any different tissue. As described by [23], the resulting distribution is the univariate non-central Fisher distribution, contained in the R-package BiasedUrn. The distribution was extended to a non-central Fisher test as described in [24] defining the p-value as the sum of the probabilities with an outcome at least as extreme as the observed one, i.e. in this case, as the sum over all those contingency tables' probabilities which have the same marginal counts (i.e. row and columns sums) as the observed event.

We assumed independence of genes in the case of the strict network. This assumption is reasonable in this context and commonly applied. (Remark D in [25] gives an account of to what degree this hypothesis is justified.)

For the 1-extended networks, we performed Monte Carlo sampling of overlap sizes by drawing 10^4 ^samples in the associated strict network and subsequently applying the strict-to-1-extended mapping procedure to each single one of these samples. This procedure gave the matrices which were taken as the basis for the pathway over-representation analyses of the extended networks. Monte Carlo p-values were calculated as done in [26] (where they are called empirical p-values; see the same article also for more references on the different ways of calculating them). Their convergence for our sample sizes was verified. A one-tailed test was used in order to test for over-representation only rather than for over- and under-representation simultaneously.

For comparison, the filtered networks were tested for over-representation of pathways using the usual Fisher's exact test against the respective reference network as background set; results were corrected for FDR as described below. The tests' source codes are included in Additional file 2.

Also for comparison, gene set enrichment analysis (GSEA, [27]) was performed. The first way this was done relied on the pure expression sets as such, the results can be found in the supplementary data, Additional file 1. The second was to perform GSEA on the reference as well as on the filtered networks with respect to the canonical pathways of the MsigDB, namely the BioCarta, the genmap and the KEGG pathway sets as gene sets. We limited the expression data set to those entries which could be linked to the filtered or reference networks' vertices mapped form the ortho-level to gene symbols, and performed GSEA with standard parameters while permuting the gene set rather than the phenotype.

#### False discovery rate control

For all tests, correction for multiple hypothesis testing was necessary. Multiplicity issues arose by performing hypothesis tests for each one out of several pathways or tissues. For this purpose, we used the method developed by (FDR) [24] controlling the positive false discovery rate as measured by q-values. As written, Storey's methods only apply to the case of continuous test statistics, where the p-values are equidistributed. However, the p-values computed from overlap sizes are discrete-valued. Indeed, this is not a problem since discrete p-values are always greater than corresponding continuous p-values which fully exhaust the level. The q-values are monotone in the p-values, thus for discrete p-values they are never too liberal and FDR control is achieved.

#### Key node analysis

The pairwise disconnectivity index (*Dis*) [15] is a vertex scoring defined as the fraction of nodes of the whole network that become disconnected after the removal of a given vertex. As such, it is a "non-local bottleneck" or "non-local degree" measure.

Each filtered network was compared with the reference network by plotting the filtered node's normalized *Dis *against the corresponding value in the reference network. In order to describe the far-from-diagonal outliers, we defined the *Dis*-increase of a vertex *v *as

$$\Delta Dis\left(v\right)=\frac{Dis_{\text{filtered}}\left(v\right)}{\max_{w}Dis_{\text{filtered}}\left(w\right)}-\frac{Dis_{\text{reference}}\left(v\right)}{\max_{w}Dis_{\text{reference}}\left(w\right)},$$

obtaining the increase resp. decrease of a vertex' influence in the filtered compared with its influence in the reference network.

Orphan vertices were discarded due to the fact that removing vertices without edges yields no changes in the connectivity of the network. Additionally, we performed GO-term over-representation analysis with Onto-Express [28,29] on the metabolic networks.

Differential expression values were tested for equal variances using Levene's test [30] due to its aptitude for symmetric, moderate-tailed, distributions [30].

Biological functions were retrieved from TRANSPATH unless mentioned otherwise.

## Results

### Mapping

The basic data which underlies the procedure is a reference network. Here, we chose to consider signaling network derived from the TRANSPATH database by the "semantic network syntax" [17]; this means that an arrow stands for a process of active forwarding of information, a typical edge A **→ **B may therefore stand for the process "kinase A phosphorylates protein B". The metabolic reference network is the KEGG network with enzymatic activities as nodes. Each reference network was filtered with the SAGE breast cancer (BC) expression data from CGAP. In turn, the resulting networks were considered in two variants. The first one, called strictly filtered network, is the set of detected and mapped protein identifiers, while the second one includes their in- and out-neighbors in the underlying reference network. In both cases, all TRANSPATH resp. KEGG edges were drawn between the chosen vertices. We used the union of normal and disease sample sets in order to treat both conditions in a unified framework, retaining the information on the difference sets (the union minus the intersection) for detailed analysis on a case-to-case basis. This approach has the advantage of minimizing fragmentation and maximizing the number of vertices. For instance, we have 183 weakly connected components in the disease sample's strictly filtered network (585 vertices), 190 in the normal tissue sample (606 vertices), 168 in the strict union (609 vertices) but 43 in the 1-extended union (1410 vertices). Another reason for choosing the union rather than the normal resp. disease set or the intersection was to prioritize tissue- over disease-specificity. The symmetric difference set (i.e. the set of nodes detected in either disease or normal tissue but not in both) of the breast cancer networks was found to be relatively small.

For over-representation analyses, the whole vertex set including the orphans was used, whereas the orphans were discarded for topological analyses.

The sizes of the resulting networks are given in Table 1 ranging between 170 and 609 nodes with an average degree between 1.2 and 1.3 in the first and 1.82 in the second filtering step. The numbers of vertices of the 1-extended networks are between 2.3 and 6.4 times those of the strict ones.

**Table 1:** The filtered networks' sizes.

Network	Data	Strict			1-extended		
		**# V**	**# E**	**# WCC**	**# V**	**# E**	**# WCC**

Signaling	Ref.	1843	3931	54			
	
	Filtered	609	770	168	1410	2564	43

Metabolic	Ref.	1793	5711	21			
	
	Filtered	172	225	71	1091	1983	16

This table shows the two reference networks (Ref.) and their filtered sub-networks with their number of vertices (# V), number of edges (# E) and their number of weakly connected components (# WCC). The expression data was obtained from experiments comparing breast cancer with normal breast tissue. The number of weakly connected components is reduced drastically between strict and 1-extended filtering.

### ExPlain

It is of interest to assess to which extent tissue-specificity is retained in the filtered networks, due to possible information loss inferred by the mapping procedure. The ExPlain tool [21] is the only one designed for this purpose and appropriate for the TRANSPATH gene identifier format. However, the ExPlain and tissue analysis was only performed on the signaling network; a similar analysis of the metabolic network would be severely hampered by an unavoidable explosion of the numbers of genes due to the fact that enzyme identifiers are linked to a huge set of genes, which would render any over-representation analysis virtually meaningless.

It turned out that in most cases the sample tissue or tissues in their anatomical proximity can be recovered. In fact, ExPlain confirmed the mammary origin in four out of the six cases given by the three top-ranked networks in each of the two signaling networks, and inferred lymph tissue characteristics in the 1-extended case.

Likewise, tissues of the mammary gland occurred, showing that also the 1-extended filtered network retains very tissue-specific information. Moreover, the male reproductive organs figured among the least significant tissues in both networks with *p *> 0.5 which may be seen as a negative control (data not shown).

Additionally, we found that the most over-represented disease in both networks is breast neoplasms (*p *= 2.8 × 10^-27 ^in the strict, *p *= 1.8 × 10^-44 ^in the 1-extended, ExPlain disease category over-representation analysis).

### Pathways

Over-representation analysis was carried out on grounds of TRANSPATH and KEGG pathways as underlying databases. We tested the resulting strict and 1-extended networks' gene sets for pathway over-representation against the null hypothesis that genes are drawn at random from the reference network with probabilities which correspond to overall expression values across all tissues, i.e. we consider the situation where it is more likely to draw a ubiquitously expressed gene such as beta-actin than to draw a highly specialized one such as alpha-synuclein. A significant test result will then imply the presence of highly specialized genes.

For the strict case, samples are drawn without replacement from the pool of reference vertices with probabilities given by the portion of expression level, averaged over all tissues, among all expression averages of the reference network, leading to a test whose null distribution is the non-central hypergeometric distribution [23], in contrast to the central one occurring in Fisher's exact test. For the 1-extended network, dependence of the genes caused by the construction is accounted for by means of Monte Carlo sampling of the resulting 1-extended overlap sizes, leading to empirical p-values.

Since over-representation analysis would favor large TRANSPATH orthofamilies, the pathways were mapped to the reference graph identifiers, yielding 137 pathways with 48 members on average, and subsequently the latter to the human Affymetrix identifiers.

### Spindle checkpoint

**Figure 2:**
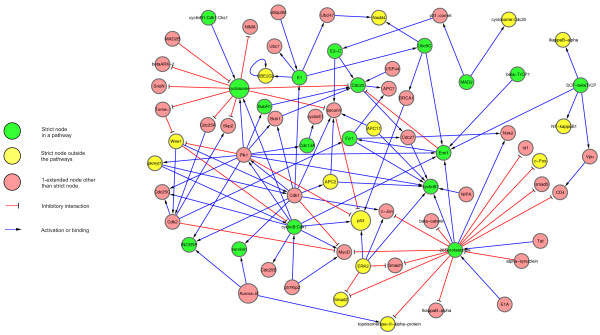
**Neighborhood of over-represented pathways in the breast cancer signaling networks**. The three significantly over-represented cell-cycle-related pathways' strict overlaps and the corresponding 1-extended nodes in the breast cancer signaling networks are connected, and a selected neighborhood of them is depicted in this figure. The pathways are "metaphase to anaphase transition", "cyclosome regulatory network" and "Aurora B cell cycle regulation". Those nodes of the strict network which belong to at least one of these pathways are depicted in green, those strict nodes which do not belong to a pathway are depicted in yellow, and those which belong to the 1-extended but not to the strict one in red. Inhibitory arcs are depicted in red, blue-colored arcs comprise non-inhibitory semantics such as activation and binding. The red nodes strongly enhance the network's connectivity. All paths from cyclosome to p53 pass at least one red node. The interaction between the three nodes Aurora B, cyclosome and p53, and thus the metaphase/anaphase-cell cycle-functional module only appears in the 1-extended network since securin, MAD2B and BRCA1, crucially involved in spindle assembly and spindle checkpoint, are not part of the strict but included in the 1-extended network.

In most cases, we found that it is possible to interpret and group the pathways by assigning them to superordinate functional categories. There were 53 pathways overlapping with the strictly filtered breast cancer signaling network in at least two vertices (see supplement). Among them, six have significantly larger overlap than predicted by the null model (q < 0.05), "Aurora-B cell cycle regulation", the "TGF-beta pathway", the "metaphase to anaphase transition", the "wnt pathway", the "cyclosome regulatory network" and the "PDGF pathway". Among those, Aurora-B, metaphase to anaphase and cyclosome are cell-cycle-related, and in fact several of the filtered networks' proteins belong to all of them. We compared those vertices which belong to at least one of these three pathways with the neighborhood in the 1-extended network (Figure 2). Several functional relationships between the strict vertices became visible only upon the network's extension. One of the core proteins regulating the metaphase-anaphase transition and exit from mitosis is cyclosome. In fact, interactions of cyclosome with other crucial cell-cycle regulators such as Aurora-B, p53 and the prominent breast cancer related protein BRCA1 could only be observed in the 1-extended network. The metaphase to anaphase transition is characterized on the molecular level by separation of the sister chromatides which is controlled by the mentioned proteins crucially involved in the spindle checkpoint of the cell-cycle. For instance, Aurora-B regulates the correct bi-orientation of the chromatides and prevents the next cell-cycle phase if the microtubules bind incorrectly [31]. In general, passing the spindle checkpoint is necessary for cell proliferation, clearly increased in breast cancer cells. In fact, inhibitors for Aurora proteins, thus inhibitors for mitosis, have been proposed to be candidates for future chemotherapies [31]. Aurora-B, BRCA1 as well as other cell-cycle regulators, such as securin and MAD2B are not part of the strict but included in the 1-extended network. In general, the complex interactions of crucial pathways in the reference network resolved into a manageable situation in the strictly filtered one while the principal molecular agents are completely listed in the 1-extended one. Moreover, the clear interactions of the mentioned pathways were obscured in the reference network by the high connectivity of p53 with 20.94% of all reference vertices being reached in its 2-neighborhood (defined by applying the extension procedure twice), cyclosome (65 vertices in the 2-neighborhood) and ERK2 (339 vertices in 2-neighborhood).

Monte Carlo pathways over-representation analysis yields that the two over-represented pathways of the 1-extended breast cancer network are the "cyclosome regulatory network" and the "alpha IIb beta3 pathway" having both the maximal overlap. Receptors of the integrin family are on top of signaling cascades targeting proliferation, differentiation and apoptosis. Thus, the analysis of the 1-extended network added the valuable piece of supplementary information.

The situation in the strict metabolic network case was similar, with the set of significantly over-represented pathways being very small. Only "chondroitin sulfate biosynthesis" and "sphingolipid metabolism" were significant. The chondroitin sulfate forms "melanoma-associated chondroitin sulfate proteoglycan" involved in cell motility of cancer cells [32].

The second pathway shows consistency with the signal transduction network analysis, allowing for analysis from an integrated viewpoint. In fact, sphingolipids gain their pro-and anti-survival effect by activation of ERK1/ERK2 [33,34].

Passing to the 1-extended metabolic network, seven pathways were significantly over-represented with respect to the 1%-significance level. These pathways can be grouped into three functional categories, namely signal molecule biosynthesis ("sphingolipid metabolism" and "chondroitin sulfate biosynthesis"), energy metabolism-related pathways ("starch and sucrose metabolism", "glycolysis/glyconegenesis"), and essential metabolite-producing pathways ("Methionine metabolism", "pantothenate and CoA biosynthesis", "pyrimidine metabolism").

For comparison, GSEA yields only two enriched pathways, namely "glycolysis" (q = 0.026) and "gluconeogenesis" (q = 0.042) on the genmap gene set for the BC phenotype (supplementary data), restricting the expression data to the metabolic reference network's nodes. The detailed result sets are included in Additional file 3.

### Key nodes

In this section, we aim at the extraction of biological insights in the form of predictive information on the networks' key nodes, defined as those vertices whose knock-out is predicted to influence the network's functioning in the most harmful way. This concept is made precise by the Pairwise Disconnectivity Index, henceforth called *Dis*, established by [15]. It measures the fraction of connected pairs of nodes which become disconnected after the node's removal. As our main focus is on filtering, we shall also be interested in the increase in a node's disconnectivity inferred by passing from the reference to the filtered network (see Table 2).

**Table 2:** Summary of the disconnectivity key node analysis.

Network	Method	1^st ^Key node	2^nd ^Key node	3^rd ^Key node	4^th ^Key node	5^th ^Key node
Signaling Network	strict	MEK1 (0.21)	SHP-2 (0.20)	Abl (0.18)	ERK1 (0.14)	MEKK1 (0.13)
	
	1-ext.	p53 (0.16)	SHIP (0.06)	PIAS1 (0.06)	Fyn (0.06)	PAK2 (0.04)

Metabolic Network	strict	Nucleoside-diphosphate kinase (0.39)	Hexokinase (0.32)	Phosphatidate phosphatase (0.26)	Glucosyl-ceramidase (0.25)	Glucosyl-ceramide synthase (0.24)
	
	1-ext.	Lipoprotein lipase (0.22)	Nucleoside-diphosphate kinase (0.14)	Succinate-CoA ligase (GDP-forming) (0.14)	5'-nucleotidase (0.12)	Hexokinase (0.11)

The five *Dis*-top-ranked vertices are shown for each filtered network. Most prominent vertices in the signaling networks are related to the "EGF network". The proteins hexokinase, lipoprotein lipase and further high-ranking nodes of breast cancer metabolism indicate that energy supply and conversion play the major role.

**Figure 3:**
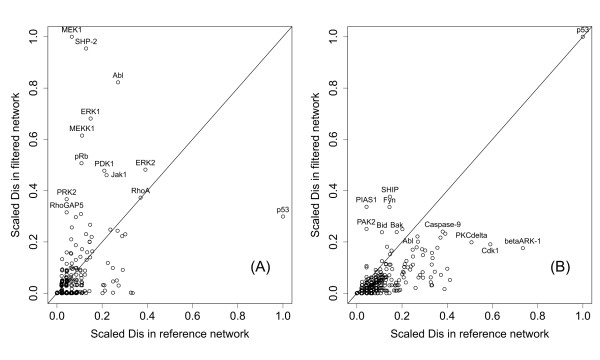
**Comparison of the reference and filtered networks' disconnectivity**. A vertex' value indicates the portion of all connected pairs of vertices in the network that become disconnected after its removal. For better comparison, the values are normalized to fill the interval [0, 1]. **(A) **The scaled *Dis *in the breast cancer data strictly filtered network is plotted against the scaled *Dis *in the underlying reference network. EGF pathway-related kinases increase while p53 drastically decrease. **(B) **In the corresponding 1-extended network, a large cluster of nodes has small *Dis *before and after filtering, only p53 remains at the highest level.

**Figure 4:**
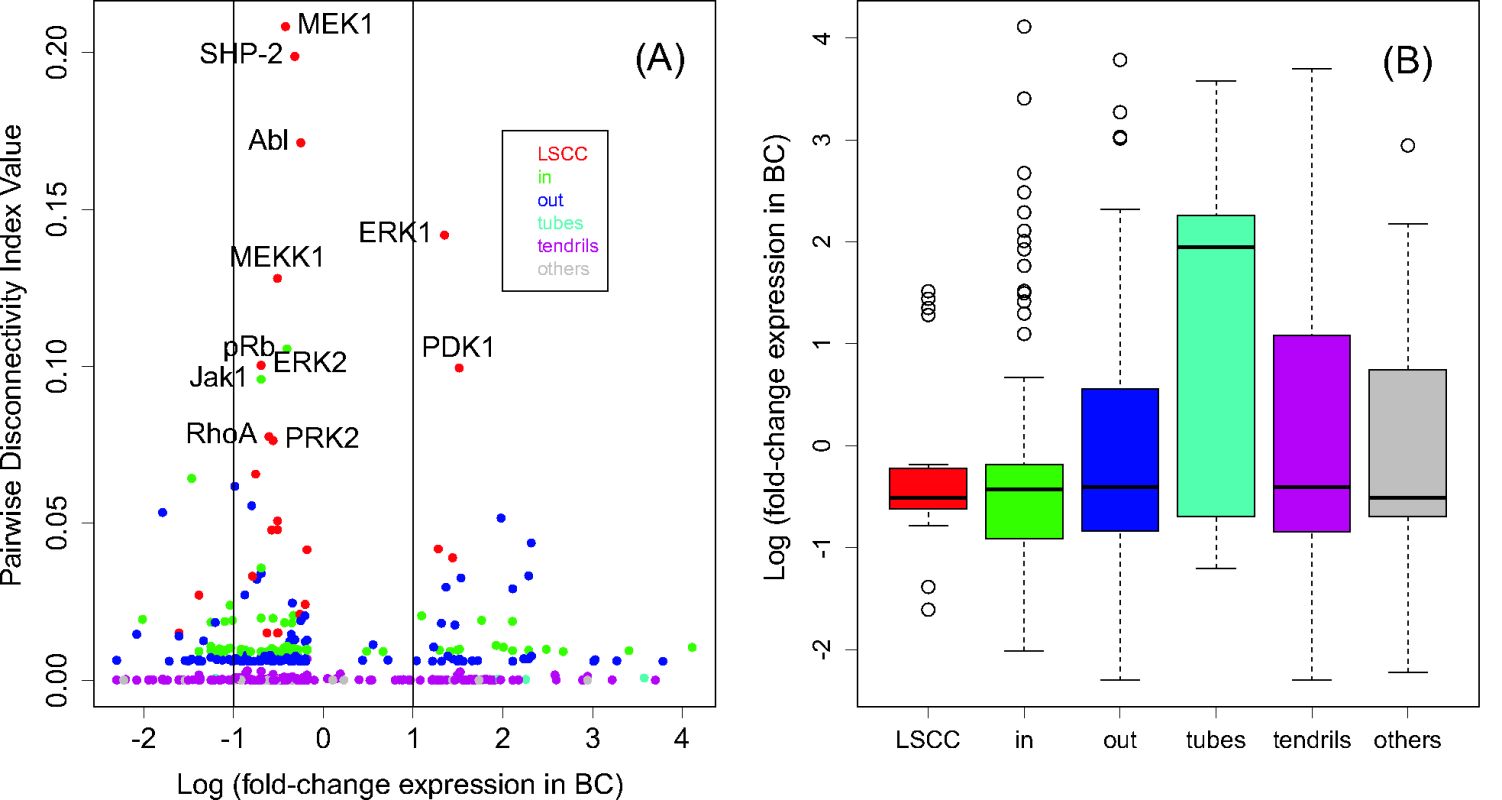
**Plot of logarithmic fold-change expression versus pairwise disconnectivity index, colored by membership to a compartment in the bow-tie model of the strictly filtered breast cancer signaling network and box plot of the numbers of differentially expressed tags in the breast cancer case, binned by bow-tie compartment membership**. **(A) **The plot for the strict breast cancer signaling network. Remarkably, most nodes with high disconnectivity have small log fold-change between -1 and 1 (vertical lines). These are ubiquitously expressed genes with high importance for network connectivity. However, there are two exceptions, ERK1 and PDK1, the phosphorylators of RSK. Almost all topologically prominent nodes belong to the largest strongly connected component (LSCC). Moreover, different colors, corresponding to the bow-tie components, show different variability of differential expression. This motivates the next plot. **(B) **Topologically central nodes are in fact more stably expressed. Logarithmic ratio of number of tags between normal and disease tissue was plotted with the same colors as in (A). Variances differ clearly between groups implying a correspondence between topological features and expression: Central molecules, i.e. those of the LSCC, have strong tendency to be not differentially expressed, showing that these are molecules which are essential to the cell's functioning.

The nodes with the highest *Dis*-increase turned out to often belong to over-represented pathways. In the signaling network (see Figure 3), most of the nodes with highest *Dis*-increase between the reference and strict situations were part of the EGF pathway, with all of its kinases, namely MEK1, ERK1, ERK2, Src, PKC and PDK1, appearing among the first eight entries with respect to *Dis*-rank. Thus, the EGF pathway plays a crucial role, detected by topological features rather than by mere over-representation. The second-ranked vertex SHP-2 shares with EGF's ErbB1 and Grb-2 the close common downstream molecule RAS. Additionally, it is part of the PDGF-pathway which has been identified as the sixth-ranked over-represented pathway. Remarkably, members of the top-ranked pathways, especially those of the "metaphase to anaphase transition" have small or even decreasing *Dis*, which is explained by the fact that none of its members belong to the bow-tie-model's LSCC which tends to assemble the nodes with high *Dis *(Figure 4A).

In total, the PDGF and EGF pathways' impacts on the breast cancer networks added to pathway analysis. The scatter plot of the scaled disconnectivity values of the 1-extended network differed drastically from the one in the strictly filtered network (Figure 3) in the sense that they form a large cluster consisting of nodes with small *Dis *before as well as after filtering, and a distinct outlier, the transcription factor p53. This is explained by the fact that on average single nodes have lower overall disconnectivity weights in large networks while adding all neighbors of p53 increases its influence.

The plot of differential expression against disconnectivity (Figure 4A) shows that the central molecules, i.e. those with highest disconnectivity, remain stably expressed or induced in cancer with the exception of ERK1 and PDK1, both up-regulated in breast cancer. In fact, it has been described that both kinases regulate RSK1 activation through distinct events; RSK1, in turn, regulates proto-oncogene and transcription factor c-Fos. Both phosphorylations together have been shown to be required for full activation of RSK1 [35].

Furthermore, visual inspection of Figure 4A suggests that the amplitude of variation of differential expression varies between the compartments of the bow-tie model; in particular, differential expression of the in-components seems to be larger than that of the LSCC (Figure 4B). This is confirmed by the Levene test for inhomogeneity of variances, p = 0.01615. Thus, there is a remarkable correlation between topological features of the filtered signaling network and differential expression: Constituents of the LSCC are more vital for cellular functioning, and hence more stably expressed than the tendrils.

Expanding the signaling network to the 1-extended filtered one, the set of high-ranked nodes fell into two functional categories, an apoptosis-related one and an EGF-related one (Figure 3). The latter consisted of the proteins SHIP, PIAS1, Fyn, PAK2 and ERK2 [36,37].

Thus while the EGF network's constituents themselves appeared in the strictly filtered network, some positively and negatively acting crucial regulatory elements on top of it emerged in the 1-extended network's analysis. The apoptosis subset was made of p53, Bcl-B, Caspase-9, Bak and Bid. Among these, p53 and Caspase-9 are well-known apoptosis regulators, while Bcl-B and Bid are biomarkers for frequent cancers such as breast neoplasms [38]. Those members of the apoptosis group which belonged already to the strict network showed a drastic *Dis*-increase between strict and 1-extended; similarly, the apoptosis-related functional group of over-represented pathways mostly increased in significance-ranking between strict and 1-extended (Figure 3).

The top ten *Dis*-high-ranked enzymes of the strictly filtered metabolic network can be clustered into four groups, namely purine and pyrimidine metabolism, energy metabolism, sphingolipid metabolism and fatty acid metabolism.

The members of top-ranked pathways were recognized by pairwise disconnectivity analysis as a core component of the strictly filtered breast cancer metabolic network's cancer-affected metabolism.

All top-ranked and top-increased vertices of the 1-extended network clustered in purine and pyrimidine metabolism, energy metabolism or fatty acid metabolism.

## Discussion

Construction of the reference network and its mapping properties are crucial for subsequent analysis. We could show that the TRANSPATH signaling network provides the right compromise between coarseness and specificity. The KEGG-derived network did not have this well-balanced property, leading to difficulties in large-scale interpretation.

Network validation is strongly dependent on theoretical tools. However, it seems that so far no such tool has turned out to provide the gold standard for this purpose. The tools at hand did not recognize in all cases the initial expression data's origin (tissue resp. disease) to a satisfactory level of significance. Although it is doubtlessly not possible to recover all characteristics of the initial data, we determined a reason for the limited analytical power of the tools at hand to be related to a problematic choice of the null hypotheses. This was shown by the lack of over-represented pathways in the Fisher's exact test-analysis (supplementary data) involving the hypergeometric distribution as the standard null distribution with the drawbacks described above. Here, we proposed two different approaches. First, we proposed for each the strict and 1-extended networks a test for over-represented pathways based on an adjusted null distribution. Therefore, we replaced in the strict case the standard Fisher's exact test by a non-central Fisher's exact test, in order to include the expression levels of the present and non-present genes. This test performed better than the standard test in terms of smaller p-values and biological interpretability (supplementary data).

There are a few obstacles for comparison with GSEA. First, GSEA's focuses on the identification of enriched sets on the basis of differential gene expression. Subramanian et al. explicitly mentioned [26] that "GSEA considers all of the genes in an experiment, not only those above an arbitrary cut-off in terms of fold-change or significance". In contrast, our approach is deliberately non-quantitative-we are merely interested in presence or non-presence. The reason is that for the signaling network even small but hard-to-detect changes in expression may have impact on transduction, and for the metabolic network that we are interested in the enzymes whose expression is not well correlated with reaction rates. Moreover, there is some difficulty in relation with gene identifier mapping. In fact, the signaling networks are given in terms of TRANSPATH identifiers and these would have to be mapped backwards to UniGene identifiers, inferring a considerable loss of specificity. Moreover, the data obtained from CGAP is rather unsuitable for GSEA analyses since the samples' expression values are pooled, leading to class permutation being impossible. In general, it is hard to compare our pathway analysis methods basing on network membership and additional expression information, with a tool implementing different statistical tests on qualitative gene expression data. Thus, information from GSEA was hard to interpret; moreover, there were few significant gene sets. This may also be explained by the fact that GSEA relies on differential expression; however, most of the strictly filtered signaling networks' members were not differentially expressed (see Figure 4).

All proposed tests show that it is well feasible to develop approaches to overcome the drawbacks of the standard tools. The problem of correction for multiple hypothesis testing turned out to be important even for the relatively small numbers of tests. The standard procedure for this purpose is the Benjamini-Hochberg correction. However, recent literature points out that the BH procedure is in many situations too conservative and suggests replacing the control of the false positive rate performed by the p-values by that of the false discovery rate performed by q-values. A technical issue concerns the problem of the statistical dependency of different tests, meaning that it is hard to take account for the fact that often test statistics are correlated. For instance, overlap counts with different pathways are statistically dependent whose impact on the multiple hypothesis testing problem is nearly impossible to estimate; in particular it is unclear to what extent Benjamini and Yekutieli's notion of positive regression dependency is applicable [39]. However, the problem has already been recognized and discussed in the literature [24].

There are some recently proposed approaches to reconstruct networks combining database knowledge with expression data, namely for instance Cytoscape [40] or TS-REX [14]. Nevertheless, our proposed gene expression analysis work-flow differs fundamentally from the one implemented in these tools. Cytoscape is designed as a platform enabling the user to create plug-ins to manipulate and visualize networks by attributing external data to its entities. TS-REX scopes at reducing false-positive edges in a predicted TF-Network by using tissue-specific expression probabilities derived from EST libraries and known interactions from the TRANSFAC database. In contrast, we focus on reliable database-derived networks limited to expressed genes and their neighbors. We presented a set of consistent analysis methods to identify the filtered networks' core components.

The pipeline we have described is rather sensitive to the choice of the underlying gene set; first, one has to find a detection threshold, second, there is the ambiguity which samples of the expression data, i.e. their intersection, union or both are preferable. In our cases, we chose the union of both samples each, however, any set of genes can be applied in our approach. For SAGE data it clearly lends itself to use all genes whose corresponding tags occurred in the resulting set. Whenever another type of data source, for instance microarrays, is used, an appropriate way of classification of active genes has to be considered.

Mapping of gene identifiers, being a potential source of ambiguity, was resolved due to the good cross-links to the used expression data's TRANSPATH identifiers, while the mapping possibilities of the KEGG-Ligand database were sparse, resulting in smaller strict networks. For instance, whenever an enzyme activity is linked to many genes, it is often unclear whether all genes are necessary for building this enzyme, all genes encode for different proteins with the same enzymatic activity, or something in between.

Subsumingly, we obtained better and biologically more meaningful results in the analysis of the TRANSPATH-derived signaling network than in that of the metabolic one.

The most satisfactory and meaningful information could be extracted from the filtered networks whenever it was possible to find common entities between pathway analysis and the key node set. For instance, both analyses hinted independently to cell cycle regulation in the signaling networks. Passing from metaphase to anaphase, i.e. the spindle checkpoint, was the most significant recurring molecular event hinted at by pathway analysis, whereas most of the nodes from the EGF network were among the top-ranked and top-increased ones. Likewise, the metabolic networks comprised sphingolipid metabolism as well as energy producing metabolism within its significantly over-represented pathways and key nodes.

## Conclusions

We have shown that in the filtered signaling networks cell cycle regulation plays an important role. Therefore, we suggest to turn experimental attention to those genes whose vertices showed the highest *Dis*-increase and are part of cell cycle-related pathways with a view toward their drug target potential. In fact, vertices showing a very high *Dis*-increase have small influence on the reference network's topology, indicating that their inactivation might have less side effects than vertices with high *Dis *but small *Dis*-increase.

We could demonstrate a tendency of differential expression to happen off the topologically central parts of the networks. Key node interpretability is increased when combining their scoring value with expression data.

The procedure can be easily adapted for further analysis with other networks and other data. In any case, it should be advantageous not to focus on just one type of reference network in order to find the most consistent elements of the underlying disease by integration of signal-transductory and metabolic aspects.

It is necessary to explore the extent to which the proposed pipeline can be synthesized with the numerous non-prior knowledge based approaches in microarray analysis and network inference. For instance, it should be possible to extend the mapping procedure to use Bayesian networks as the reference network or the starting point for validation. Due to the binary nature of data-based derived networks, it would clearly be beneficial to integrate the Bayesian networks' quantitative information.

## Availability and requirements

The programs which are available in the Additional file 2 are written in Perl (v5.10.0) and R (2.7.1). The Perl scripts require local implementations of the TRANSPATH and KEGG database and the BioGPS U133GNF1B.gcrma table.

## Competing interests

The authors declare that they have no competing interests.

## Authors' contributions

EW conceived the initial idea and participated in coordination. MF developed the validation methods and supervised the research. MF and MA jointly designed, developed and implemented the algorithms and wrote the manuscript. All authors have read and approved this manuscript.

## Supplementary Material

Additional file 1 **Data collection (compressed)**. This zip-file contains the networks and the background data. Further information is given in a README file. The TRANSPATH derived signaling network's adjacency list is excluded due to license agreements. Click here for file

Additional file 2 **Code collection (compressed)**. This zip-file contains the code for all pathway over-representation tests. Further information is given in a README file. Click here for file

Additional file 3 **Summary of the pathway over-representation analysis (compressed)**. For each pathway analysis, the corresponding Fisher's exact p- and q-values are included. GSEA was performed on the pure expression data set as well as those subsets of the gene identifier which could be mapped to the reference networks' or the filtered networks' vertices. Further information is given in a README document. Click here for file
